# Towards Analysis and Optimization for Contact Zone Temperature Changes and Specific Wear Rate of Metal Matrix Composite Materials Produced from Recycled Waste

**DOI:** 10.3390/ma14185145

**Published:** 2021-09-08

**Authors:** Aydın Güneş, Emin Salur, Abdullah Aslan, Mustafa Kuntoğlu, Khaled Giasin, Danil Yurievich Pimenov, Hayrettin Düzcükoğlu, Ömer Sinan Şahin

**Affiliations:** 1 Department of Mechanical Engineering, Abdullah Gül University, Kayseri 38080, Turkey; 2 Metallurgical and Material Engineering Department, Technology Faculty, Selcuk University, Konya 42130, Turkey; esalur@selcuk.edu.tr; 3 Department of Mechanical Engineering, Faculty of Engineering, Selcuk University, Akşehir, Konya 42130, Turkey; aaslan@selcuk.edu.tr; 4 Mechanical Engineering Department, Technology Faculty, Selcuk University, Konya 42130, Turkey; mkuntoglu@selcuk.edu.tr (M.K.); hayduzcukoglu@selcuk.edu.tr (H.D.); 5 School of Mechanical and Design Engineering, University of Portsmouth, Portsmouth PO1 3DJ, UK; khaled.giasin@port.ac.uk; 6 Department of Automated Mechanical Engineering, South Ural State University, Lenin Prosp. 76, 454080 Chelyabinsk, Russia; danil_u@rambler.ru; 7 Department of Mechanical Engineering, Konya Technical University, Konya 42075, Turkey; ossahin@ktun.edu.tr

**Keywords:** temperature changes, specific wear rate, analysis and optimization, metal matrix composites (MMCs)

## Abstract

Tribological properties are important to evaluate the in-service conditions of machine elements, especially those which work as tandem parts. Considering their wide range of application areas, metal matrix composites (MMCs) serve as one of the most significant materials equipped with desired mechanical properties such as strength, density, and lightness according to the place of use. Therefore, it is crucial to determine the wear performance of these materials to obtain a longer life and to overcome the possible structural problems which emerge during the production process. In this paper, extensive discussion and evaluation of the tribological performance of newly produced spheroidal graphite cast iron-reinforced (GGG-40) tin bronze (CuSn10) MMCs, including optimization, statistical, graphical, and microstructural analysis for contact zone temperature and specific wear rate, are presented. For this purpose, two levels of production temperature (400 and 450 °C), three levels of pressure (480, 640, and 820 MPa), and seven different samples reinforced by several ingredients (from 0 to 40 wt% GGG-40, pure CuSn10, and GGG-40) were investigated. According to the obtained statistical results, the reinforcement ratio is remarkably more effective on contact zone temperature and specific wear rate than temperature and pressure. A pure CuSn10 sample is the most suitable option for contact zone temperature, while pure GGG-40 seems the most suitable material for specific wear rates according to the optimization results. These results reveal the importance of reinforcement for better mechanical properties and tribological performance in measuring the capability of MMCs.

## 1. Introduction

Investigations about combining the prominent aspects of metals to obtain new and versatile materials have gained momentum in the metal matrix composite (MMC) material field [1,2]. For MMC production, several metals can be used as a matrix or reinforcement material, such as aluminum, steel alloy, magnesium, brass, bronze, and cast irons [3,4,5,6,7,8]. Bronze and cast iron, which are selected as the matrix and reinforcement materials, have received more attention than the other materials thanks to their extraordinary properties such as good surface integrity, high strength, and good tribological properties. While it is theoretically possible to combine the good aspects of two or more metals, in practice, all aspects of these metals need to be studied in detail. In previous works published by the authors, the mechanical properties (compression [4], impact [7], tensile strength [3], Brinell hardness, micro-Vickers hardness [9], etc.), machining properties [10], and microstructural analysis of MMCs used in this study have been reported. However, it is crucial to assess the tribological properties of the produced MMCs, which are directly intended for the place of use.

MMCs are designed to achieve a harmonious and good combination of various properties such as light weight, porosity, and strength [11]. From this point of view, bronze metal, frequently preferred as a matrix material in MMC systems, can be reinforced with various metals to eliminate its weaknesses and increase its performance. To overcome some performance limitations of a Cu-based matrix, different metal-based materials such as steel, aluminum, titanium and carbide, oxide, and nitride-based structures are employed as reinforcements [12,13,14,15]. A reasonable number of papers on the effects of reinforcement type and content on the bronze matrix have been published. Gronostajski and Chmura [16] examined the effect of the addition of aluminum on bronze matrix composites produced by recycling chips. They reported that ductile aluminum chips penetrate narrow zones between bronze chips. This interaction provides good structural integrity due to enhanced diffusion bonding quality between aluminum and bronze chips and resultant enhancement in the yield strength [17]. Barbosa et al. [18] reported that iron-based alloys are entirely compatible with copper alloys, and this compatibility is beneficial for improvement in the strength/hardness of copper matrix composites. Plus, they reported that the content of cast iron in the bronze matrix composites has a significant impact on strength/hardness. However, it is emphasized that the cast iron content must be adjusted according to production parameters (pressure, temperature, and time) and the usage area since increasing cast iron content up to a certain amount can cause high strength and hardness, and accompanying better structural integrity [4,9]. There is also a direct relationship between the type of metallic chips and the selection of the production process and parameters. When a cold pressing and post-sintering method is preferred, low-strength and highly porous materials can be obtained. On the other hand, fine-grained and high-strength materials can be achieved if hot pressing and hot extrusion methods are performed [19].

Considering the available studies, it is observed that various researchers have investigated the effect of different reinforcement materials and their types, contents, and several manufacturing processes on the mechanical properties of Cu-based MMCs [20,21,22,23,24,25]. In addition, it has been reported in detail that different mechanical properties, such as hardness, tensile strength, compressive strength, elongation, toughness, and fatigue life, are severely affected depending on the proposed methods. However, reinforcement materials in MMCs affect not only mechanical properties but also tribological characteristics [26]. In this regard, Wu et al. [27] reveal that adding Ti_2_SnC particles to Cu-based MMCs significantly decreases the friction coefficient and wear rate. Additionally, the wear mechanism is changed from oxidation wear to adhesive wear due to the effect of reinforcement. Gunes et al. [28] examined the effects of cast iron on weight loss and coefficient of friction values of bronze matrix composites depending on production parameters. They reported that the most dominant factor on wear is the reinforcement ratio [28]. The effects of graphene on the lubrication behavior [29], wear mechanism of graphite-copper composites [30], the surface and tribological aspects of graphene-reinforced copper matrix composites [24], microstructural and tribological evolution of MoS_2_-reinforced tin-copper-reinforced composites [31], and particle size effect on the wear performance [32] have been experimentally investigated by different researchers.

Furthermore, the two most important parameters which give essential insight into the tribological performance of MMCs are specific wear rate and temperature changes. Unlu and Atik [33] conducted a study investigating temperature changes of tin-bronze and zinc-bronze by a radial journal bearing wear test unit. They reported that the highest temperature and specific wear rate occur in CuSn10 and CuZn30 compared to pure metals. Wang et al. [34] indicated that specific wear values of MMCs can be reduced with SiC particle reinforcement. Another study reports that increasing the reinforcement material leads to improved specific wear rate values [35]. Kozma [36] investigated the effects of iron content on the tribological properties of Al-based MMCs and reported that as the iron content decreases, the specific wear rate increases.

Based on the above discourses, materials’ mechanical, tribological, and other properties, especially MMCs produced by the powder metallurgy route, are greatly affected by several concurrent factors: matrix and reinforcement type, sintering temperature, time, pressure, interfacial bonding, etc. [37]. In addition, different researchers state that there is a reciprocal relationship between specific wear rate and temperature in MMCs. It can be observed that the wear rate of MMCs decreases with increasing temperature; then, it rises with elevated temperatures [38,39]. On the other hand, temperature and wear rate can be controlled with reinforcement materials and their ratio [40]. For instance, the wear rate can be decreased or increased for the same specimen depending on the experimental parameters and reinforcement ratio [41]. Thus, the determination of a tribological characteristic of MMCs can be quite complicated. This sophistication stems from not only the tribological parameters but also production parameters (pressure, temperature, and mixture ratio) of MMCs. Therefore, statistical evaluation and optimization of the results are essential [42,43,44,45]. Taguchi [46,47], ANOVA [48,49], artificial neural network (ANN) [50], response surface methodology (RSM) [51], and grey relational analysis (GRA) [52] methods could be used in order to evaluate wear parameters in MMCs.

Considering published studies in the current literature, it was found that various studies reported the tribological properties of Cu and different metal-based MMCs. However, there was no available work about the tribological performance of recycled waste metallic chips consisting of CuSn10 and GGG-40, which are often found as waste materials in different industries. In this context, this study paves the way for the recycling of materials consisting of these and other similar systems and the use of these materials, which are obtained as a result of recycling, as plain bearing machine elements. These hypotheses are an essential point that the present study offers as a different solution to those in the open literature, which still remains a blank spot in the available works. The main subject of this paper is the production of MMCs by combining waste metal chips with an unconventional method and the determination of the tribological behavior of this material and its association with other known properties. In the present study, bronze (CuSn10) matrix reinforced with spheroidal graphite cast iron (GGG-40) composites was produced. CuSn10 and GGG-40 metallic chips were hot pressed at four different mixture ratios (90 wt%, 80 wt%, 70 wt%, and 60 wt%), three production pressures (820, 640, and 480 MPa), and two temperatures (400 and 450 °C). CuSn10, which is common in industry as waste metallic chips and is used as a self-lubricating bearing material, was chosen as the matrix phase. In addition, GGG-40 metallic chips, abundant in industry, were employed as reinforcement material to strengthen the bronze matrix. Two important wear parameters, specific wear rate and temperature changes of produced MMCs after a wear test, were experimentally measured and statistically analyzed. In addition, the findings were further evaluated using the Taguchi S/N ratio, which gives reliable results on wear test parameters for optimization of production parameters. Tribological parameters and production parameters were associated, and the best MMC specimens for each condition were examined.

## 2. Materials and Methods

**Figure 1:**
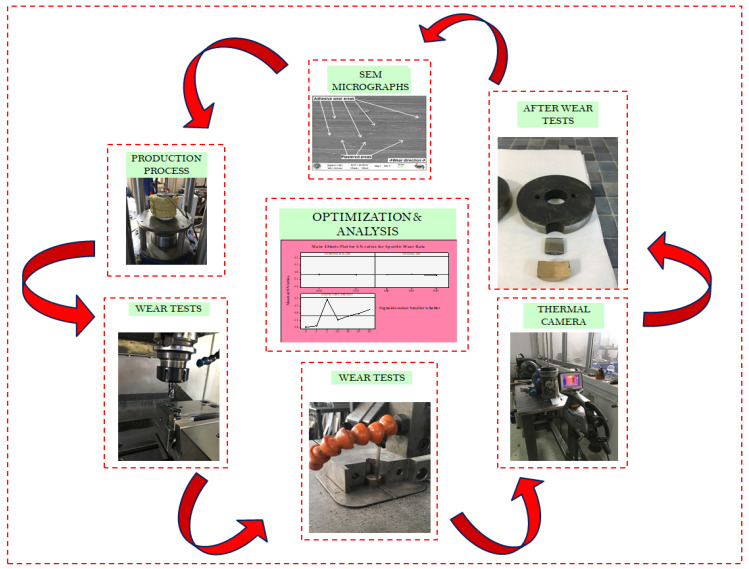
A general outline of the study.

In this study, MMCs, consisting of waste metallic chips, were produced by a double-acting hot pressing method. The chemical compositions of the reinforcement (GGG-40) and matrix (CuSn10) materials are given in Table 1. Production stages and more detailed information are available in the authors’ prior works [4,53]. The superior mechanical properties of GGG-40 reinforcement and the high corrosion resistance, superior electrical and thermal properties of the CuSn10 matrix phase, and, most importantly, their suitableness as bearing materials played an essential role in selecting these materials [28]. Plus, GGG-40 was employed as the reinforcement element due to the lubricating effect of the dense spheroidal graphite found in its structure. In this way, it was aimed to gain a significant advantage in MMC systems with self-lubricating bearing properties. The general view of this study, comprising the production process of composite materials, the preparation stages of the produced composite materials for wear tests, instruments used for wear tests, and the optimization of outputs, i.e., temperature changes and specific wear rate, is presented in Figure 1.

### 2.1. Composite Material Production Process

The double-acting hot pressing method was used to fabricate composite materials. During the production, 5 different mixing ratios, 3 different pressures, and 2 different temperatures, which are specified in Table 2, were used. Reinforcement content and other production parameters were determined by our previous knowledge, preliminary examination, and literature surveys [4,54,55]. Firstly, cylindrical CuSn10 and GGG-40 bars were machined in a conventional lathe with the same cutting parameters to produce metallic chips. Then, the differently sized metallic chips were sieved with 1–2 mm sieves, and the chips remaining between the sieves were used in this study. The metallic chips were mixed homogeneously via a double-cone mixer [56]. After the mixture process of the metallic chips, they were poured into a male mold, and they were kept for 15 min at different production temperatures (400 and 450 °C) to achieve homogeneous temperature distribution. Finally, the metallic chips were consolidated by synchronistical movement of upper and lower molds under different pressures (480, 640, and 820 MPa) for 10 min. Thus, both a homogeneous temperature distribution and the formation of the desired plastic deformation between the metallic chips can be achieved [9]. More detailed information about the utilized reinforcement and matrix materials and production process of MMCs has been reported by the authors [9,56]. The diameter of the composite materials removed from the mold was 19.6 mm, and their lengths varied from 32–36 mm depending on the applied temperature and pressure parameter. As the temperature or pressure parameter increased, their length shortened due to the decrease in the spaces between the chips.

### 2.2. Wear Tests

**Figure 2:**
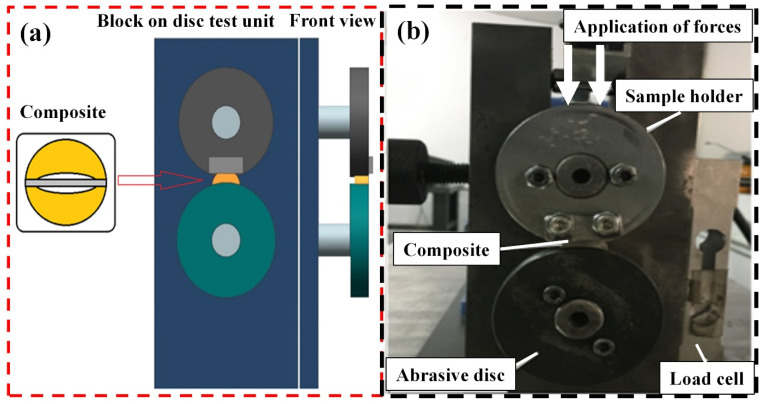
(**a**) Schematic view of the wear test setup, and (**b**) wear test machine [28].

To determine the mechanical properties of the produced composite materials, many experimental studies were carried out before the wear tests, and it was observed that the produced MMCs exhibited satisfying microstructural and mechanical properties [4,7,9]. Then, to investigate their usability as a self-lubricating bearing material, wear tests were carried out by a block-on-disc test device. In these experimental studies, the unprocessed composite materials removed from the mold were first sliced with the help of a 10 cm wide jigsaw device and then divided into two down the middle. During this division process, the gray-colored part of the composite material (as shown in Figure 2a) showed the losses after cutting with a 2 mm jigsaw. The white parts remaining in the interior region showed the losses that occurred during the formation of the bedding gaps. To ensure proper bedding in the wear tests after the cutting process, the inner parts of the composite materials were machined with the help of CNC, and 69.05 mm radiuses were formed in the inner parts. In the application of this process, an abrasive disc was a 69 mm diameter was employed, and a bedding gap of 0.05 mm was formed. During these processes, no refrigerant was used to prevent any manipulating effect on the surface structure of the composite material. Afterward, an ultrasonic bath was applied to the abrasion test specimens for approximately 180 s to remove unwanted residues from the surface [57]. In addtion, after the wear tests, the microstructural evolution of some MMCs produced by different production parameters was analyzed using a scanning electron microscope (SEM, Zeiss EVO LS 10) at 20 kV.

The block-on-disc test device shown in Figure 2b, which was designed according to the ASTM (G77-05) standard, was used to perform the wear tests. The wear behavior of the produced MMCs was monitored in a specific area rather than point contact abrasion due to the macrodimensions (1–2 mm) of the metallic chips used [33]. By doing so, the whole wear behavior of CuSn10 and GGG-40, and possible micro- or macroscale void regions, can be investigated. For wear tests, the abrasive disc speed was set at 400 rpm (1.06 m/s) under a 30 N load regarding preliminary test outcomes, and it took nearly 31 min to accomplish a total wear distance of 2000 m [28]. AISI-4140 steel with a 54–56 HRC surface hardness, obtained by cementation treatment, was used to erode the composite materials during the wear tests. During wear tests, the abrasive disc was altered for each test, and 3 samples were tested from each set to verify the accuracy of the data. On the upper part of the wear test setup, a sample holder provided the abrasion of MMCs. The sample holder was fixed, and there was a disc rotating at the bottom with a shaft powered by a 2.2 kW electric motor and a 2.5 kW speed adjuster. The movement of the disc occurred in the clockwise direction, and the horizontal forces created by the friction force were recorded instantly on a computer with the help of load cells. The ratio of the vertical force to the horizontal friction force gave the friction coefficient. These values were regularly monitored during experimental studies. The friction coefficient measurements started from the first contact of the specimen with the abrasive disc. This area may vary depending on the environment and test conditions with the further stages of wear. In addition, no sudden changes were observed in surface roughness values thanks to the constant applied load and wear rate. The friction coefficient was measured with a Squirrel brand data logger with 8 analog inputs and recorded using the Squirrel View interface program. After three repetitions, weight loss that occurred during the experimental process was determined by measuring it with the “Precisa XB-220A” Swiss-made precision scale, with an accuracy of ±0.0001 g, before and after the experiments [28,53].

### 2.3. Thermal Camera

During the wear tests, instantaneous temperature changes in the wear zone were measured by a calibrated thermal camera (FLIR Systems’ Therma-CAMTM P65). The temperature changes in the disc and MMCs were observed with the thermal camera placed directly opposite the wear zone. The vertical curve seen on the thermal camera screen shows the change in temperature from top to bottom, and the temperature changes in the upper–lower parts of the contact area were followed there. The temperature range of the thermal camera was −40 °C to +2000 °C with an accuracy of ±2% or 2 °C, and the image frequency was 60 Hz. The camera had a thermal sensitivity of 0.08 °C at 30 °C and a spectral range of 7.5–13 μm with a resolution of 320 × 240 pixels [58]. The measurement of temperatures in the thermal camera was carried out throughout the vertical line from the contact point, and the changes were observed with the graphic shown on the instrument. Then, the temperatures of the contact point for each composite material were determined and assessed.

### 2.4. Specific Wear Rate

The specific wear rates of MMCs were calculated from the following specific wear equation (Equation (1)); where *Ws* is the specific wear rate, ∆*V* is volumetric material loss, ∆*m* is weight loss, *ρ* is density, *Fn* is applied normal force, and *L* is total wear distance.

(1) $$Ws=\frac{∆V}{FnL}=\frac{∆m}{\rho FnL}$$

It can be seen in the relevant equation that there is a relationship between the density and wear rate of each composite material. The pressure created by force applied to the material surface during wear can change depending on the surface structure. Excessive pores on the abraded surfaces affect the specific wear rate; even if there is the same amount of wear, the existing pores in the structure can change the sample’s specific wear rate [59].

## 3. Results and Discussion

Results of the experiments, including experiment number, the specimen codes, inputs, and outputs, are presented in Table 3. As seen in the table, five different reinforcement contents, two different temperatures, three different pressures, and two non-waste samples (i.e., pure CuSn10 and pure GGG-40) compose the full 32 experimental lines [28] for the temperature change and specific wear rate. In this section, the microstructural evolution of samples is analyzed, and then parametric optimization is presented; lastly, ANOVA results of the specific wear rates and temperature changes are reported.

### 3.1. SEM Images/Microstructures

**Figure 3:**
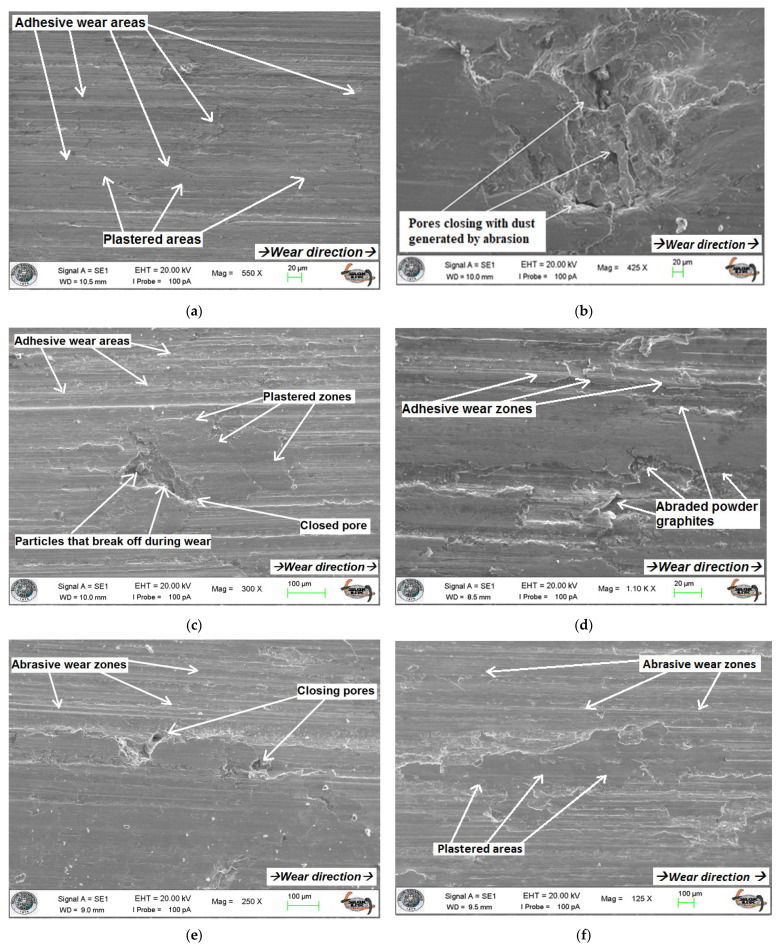
SEM images of different composites after wear (**a**) 100C, (**b**) 60C40G, (**c**–**e**) 70C30G, and (**f**) 90C10G samples produced at 450 °C and 820 MPa.

From the wear tests, the SEM images of some MMCs produced with different reinforcement contents are shown in Figure 3. To precisely observe the effect of the most dominant parameter (i.e., reinforcement ratio) on the wear aspects, these MMCs were fabricated by the same production parameters (i.e., 450 °C and 820 MPa). In the SEM images, the direction of wear is indicated from left to right, and it is observed that microstructures changed depending on the reinforcement ratio. As the reinforcement content was decreased, more adhesive wear behavior was observed in the progressive wear processes of the MMCs, as shown in Figure 3b–f. In addition, the deterioration of the surface structures caused by abrasion was less in the MMCs produced at high temperature and pressure parameters [28]. Additionally, it is seen that CuSn10 is exposed to significant plastic deformation due to the increased temperature in the surface region. Especially in the 100C sample, the plastering of CuSn10 in the direction of wear is evident (Figure 3a). Moreover, it has been observed that the porous structures in the MMCs’ structure play a vital role in reducing friction on the surface, both by closing with the wearing particles and by the lubricating effect of the eroded spheroidal graphite (Figure 3b–f). Thus, less wear occurs in the friction area, which is one of the most critical parameters that increases the lifetime of the bearing material under harsh service conditions [60,61,62].

The lubrication effect of dense spheroidal graphite in GGG-40 is also beneficial for the temperature differences in the contact area during wear. The surface structure of composites can be adversely affected due to more adhesive wear of CuSn10 with increasing temperature. However, the adhesive effect can be minimized due to the lubricating effect of GGG-40 during wear, and heat conduction can be improved in the wear area [18,63].

**Figure 4:**
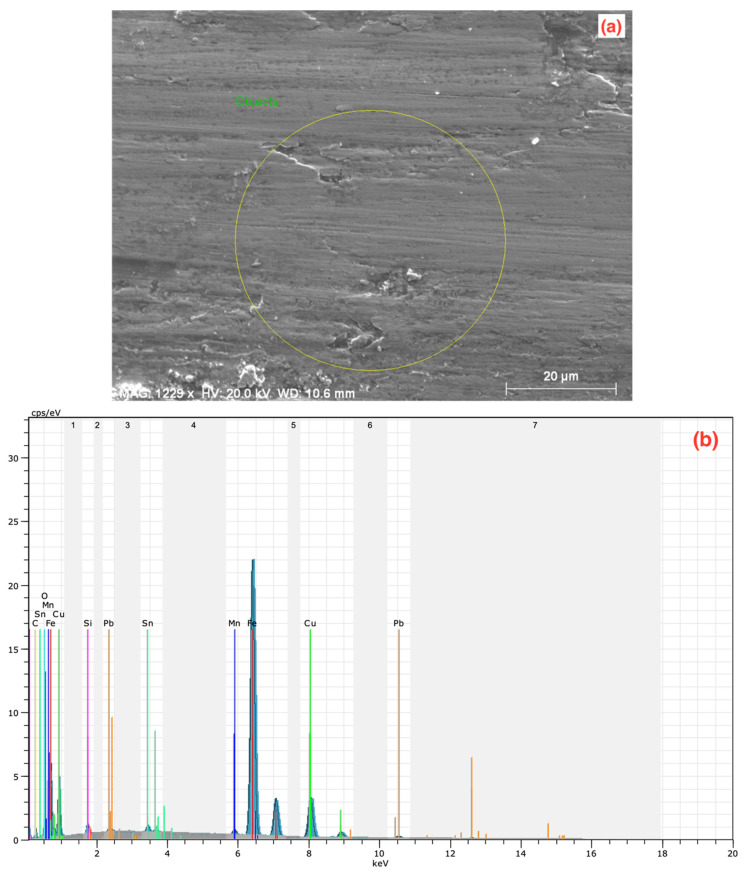
(**a**) SEM image and (**b**) EDS analysis of 70C30G composite after wear.

To better evaluate the wear behavior and surface structure of the MMCs, SEM and energy dispersive spectroscopy (EDS) analyses were performed. The SEM image of the 70C30G sample produced at 450 °C and 820 MPa is shown in Figure 4a. Figure 4b shows the elemental distribution of the corresponding region in Figure 4a (as indicated by a yellow circle). Such a large area was chosen to reflect the wear properties of the composite homogeneously.

After the wear test of the 70C30G composite, 38.60 wt% copper, 59.58 wt% tin, 2.10 wt% tin, and 2.86 wt% graphite, along with other several minor alloying elements with low contents, were detected according to the scanned area, as shown in Figure 4b. Considering EDS results, it can be said that the abrasion wear mode occurs on the surface of the abrasive disc since more iron is detected than it contains in its structure. In addition, the presence of the graphite phase is desirable in self-lubricating bearing conditions since a certain amount of this phase can provide lubrication between the shaft and the bearing during operation. In addition, the 1.83 wt% oxygen found shows that the oxidation in the environment influences the composite. The existence of oxidation is a common situation since wear tests are performed in ambient conditions. Increasing temperature in the contact area during the experiment is also effective in this formation. The detection and elimination of the oxidation’s source(s) is another crucial point that negatively affects the material’s service life [28,64].

**Figure 5:**
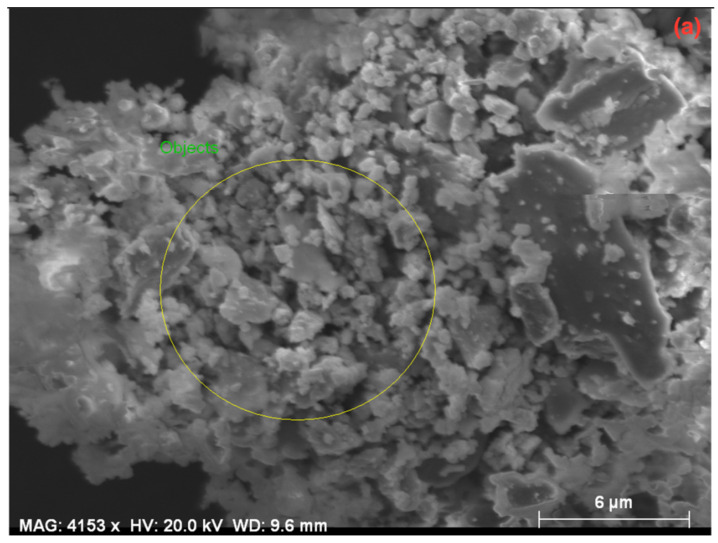

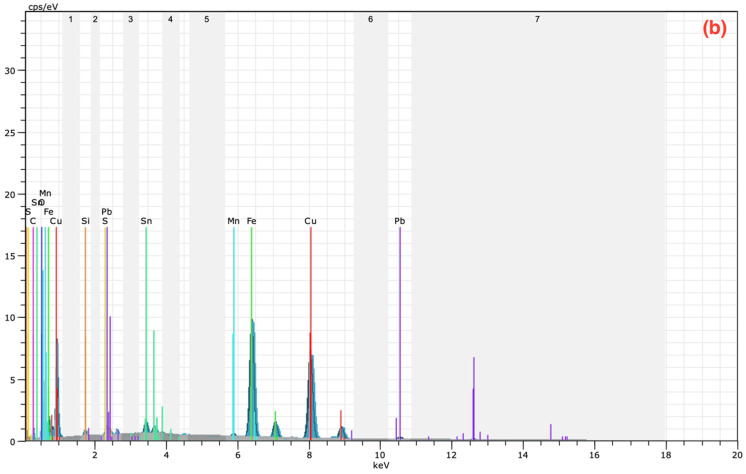
(**a**) SEM image and (**b**) EDS analysis of 60C40G particles after wear.

Furthermore, the SEM images and corresponding EDS analysis of the 60C40G composite’s abraded particles, randomly selected around the abrasion zone, are presented in Figure 5a,b, respectively. According to EDS results seen in Figure 5b, 55.80 wt% copper, 33.30 wt% iron, 5.75 wt% oxide, and 1.36 wt% graphite are detected in the abraded particles. Since these values are close to the content of the composite material, it can be deduced that the wear occurs homogeneously.

### 3.2. Interpretation of Experimental Results and Parameter Optimization for Specific Wear Rate

**Figure 6:**
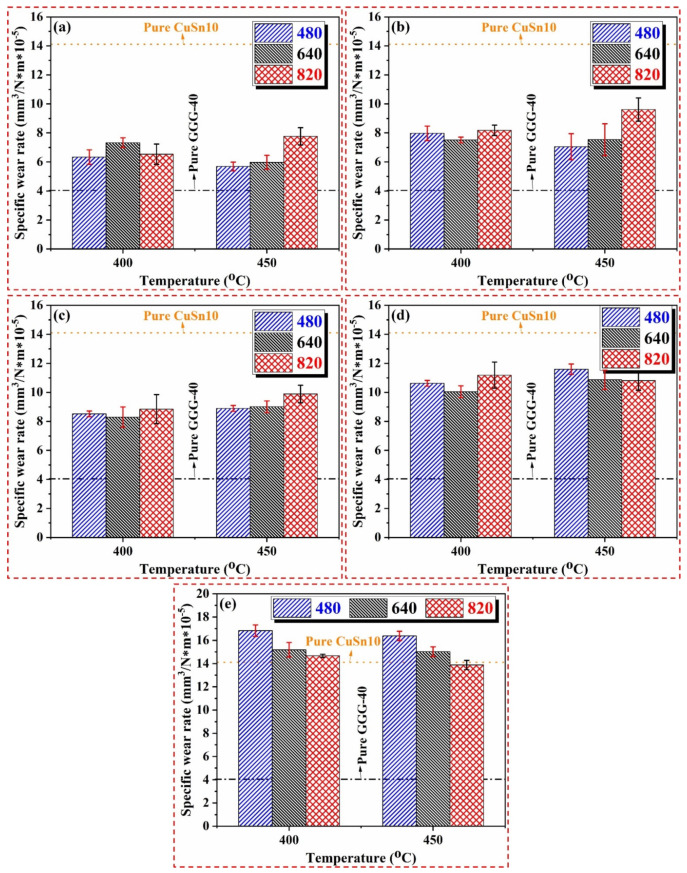
Specific wear rates of (**a**) 60C40G, (**b**) 70C30G, (**c**) 80C20G, (**d**) 90C10G, and (**e**) 100C samples with respect to different production parameters.

Figure 6 shows the specific wear rates of MMCs as a function of production parameters and sliding distance. The specific wear rate, related to the density and weight loss, was assessed according to the equation described in Section 2.4. Considering Equation (1), the pressure created by force applied to the material surface during wear varies depending on the surface structure, and it directly or indirectly affects the *p.V* factor. In addition, the pores on the worn surfaces exceeding a certain number influence the specific wear rates. In other words, the pore numbers, sizes, and shapes can affect the wear performance of the product. According to the specific wear rate results, as seen in Figure 6, the results vary significantly depending on the reinforcement content. As the reinforcement ratio increases, the specific wear rate decreases. Such a decrement is attributed to more volumetric erosion in less porous structures depending on the reinforcement content. Plus, the pure GGG-40 specimen shows the lowest specific wear rate compared to others (as shown in Figure 6) due to the initial iron chips having higher hardness than bronze chips. This situation leads to the iron chips preserving their shapes by exhibiting severe resistance to plastic deformation during the hot pressing process. The observed results in a previous work by the authors regarding the influence of production parameters on the mechanical properties [4] also confirm this case. Similar observations were also reported by different researchers [65,66] investigating the effect of porosity and reinforcement content on the tribological behavior of Cu-based MMC systems. In another study by the authors about the same material system [28], it was observed that abraded particles gradually filled the pores on the exterior surface of the MMCs with increasing reinforcement ratio, accompanying a decrement in weight loss and therefore wear rates. While the wear rates in composite materials produced at different production pressures generally exhibit similar behavior, the amount of abraded material in unit volume increases in composites produced at 450 °C, depending on the decreasing pore number.

**Figure 7:**
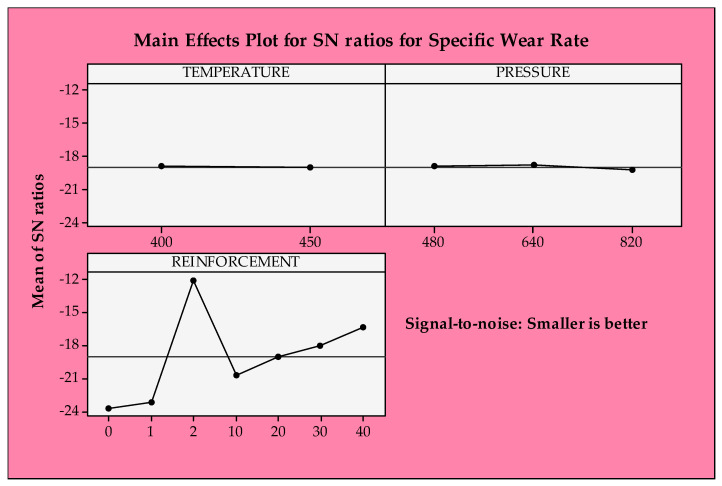
S/N ratios of specific wear rate.

Parametric optimization utilizing the S/N approach was applied to observed outcomes to verify our interpretation of the experimental results. As shown in Figure 7, the first level of temperature, 400 °C, and the mid-range of pressure, 640 MPa, appear to be the best option for the specific wear rates regarding the main plots of S/N ratios. As for the effect of reinforcement, pure GGG-40 (2) supplies the most appropriate specific wear rate conditions. After this influence, gradually increasing the reinforcement ratio from 10 to 40 wt% is beneficial for wear rate characteristics. Based on both experimental results and statistical approaches, the influence of reinforcement content is the most significant factor manipulating the specific wear rate compared to other production variables. However, the other parameters (i.e., temperature and pressure) do not exert such an apparent effect on the wear rates. As mentioned regarding ANOVA results, the production parameters, namely temperature and pressure, have no discernible effects on the specific wear rates. Statistical results support experimental interpretations.

### 3.3. Interpretation of Experimental Results and Parameter Optimization for Temperature Change

During the wear tests, the temperature changes in the contact area were instantaneously monitored via the thermal camera. Temperature change curves were obtained by recording each one-minute interval during the 31 min test period. For the temperature measurements of the thermal camera, the emissivity value was chosen as 0.7 according to preliminary examination and literature surveys [4]. Temperature variations in pure CuSn10 and GGG-40 materials are also presented in graphs.

**Figure 8:**
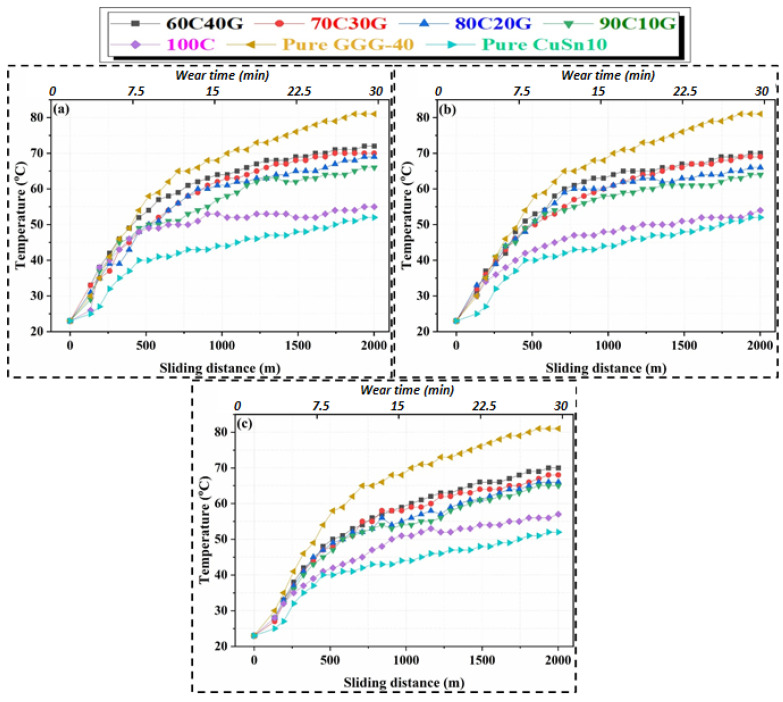
Contact zone temperature changes of (**a**) 480 MPa, (**b**) 640 MPa, and (**c**) 820 MPa MMCs produced at 400 °C according to sliding distance and wear time.

Variations in the temperature are generally related to the thermal conductivity coefficients of materials. The temperature value reached during operation in materials with a high thermal conductivity coefficient is relatively low compared to materials with a low thermal conductivity coefficient. Using the curves in Figure 8, the minimum temperature (52 °C) is observed in pure CuSn10 material, which has higher thermal conductivity, while the maximum temperature is measured as 82 °C for pure GGG-40. These values measured by the thermal camera are defined as the average temperature of the contact zone. The temperature values, which are instantaneously reached during wear and described as the flash temperature, are considerably higher than the average temperature values of this contact zone. This causes the bronze to exhibit a significant amount of adhesive behavior in the later stages of wear and finally to plaster the bronze to the surface [24,61,67].

The temperature distribution curves of composites produced at 400 °C and three different pressures (i.e., 480, 640, and 820 MPa) are shown in Figure 8. Depending on the reinforcement ratio, the temperatures of composite materials generally display a steady-state trend after 25–26 min throughout the wear test. In general, the achievement of better structural integrity in the MMC structure with decreasing reinforcement content, which has been described in detail in our previous studies [4,9,28], and the improvement of heat conduction, reduces the temperatures at the contact point. The maximum temperature values for MMCs produced at a 400 °C sintering temperature are 69 °C and 70 °C for 60C40G and 70C30G, respectively. However, the temperature value of 90C10G composite is measured as 54 °C. This remarkable change is similar to the results of our previous study [28], which examined the effect of production parameters on the friction coefficient; depending on the amount of reinforcement material, the increase or decrease in the friction coefficient affects the temperature of the contact zone. Rougher surface quality, which increases the friction coefficient, also elevates the temperature of the contact zone. Different studies [67,68] have reported such an interaction between surface quality and coefficient of friction.

**Figure 9:**
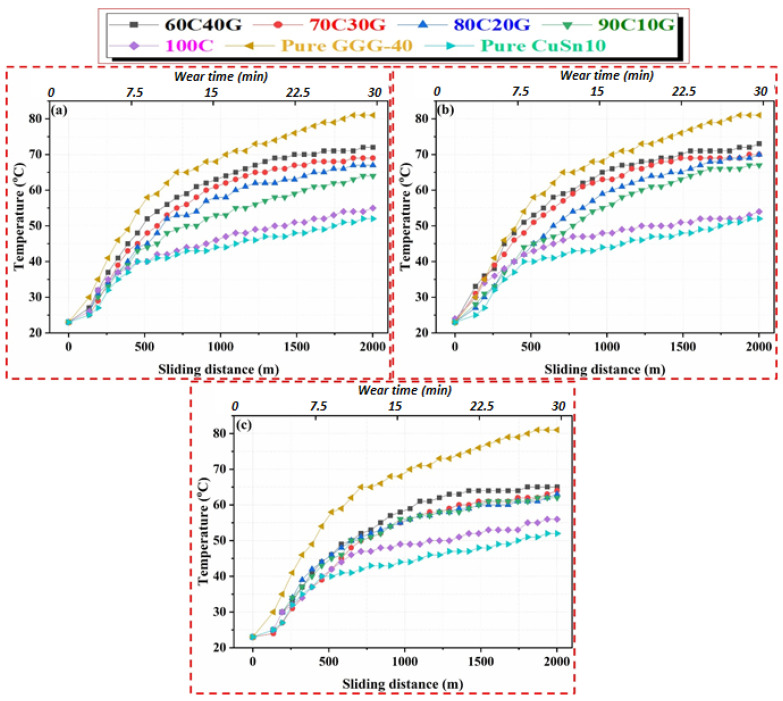
Contact zone temperature changes of (**a**) 480 MPa, (**b**) 640 MPa, and (**c**) 820 MPa MMCs produced at 450 °C according to sliding distance and wear time.

On the other hand, Figure 9 shows the temperature curves of the MMCs produced at 450 °C with different reinforcement ratios and production pressures. The changes in the temperature entered a stable region during the wear tests after approximately 20 min, and no observable change was found. As can be seen in Figure 9c, this period decreases to 15 min at a 820 MPa production pressure due to the achievement of better consolidation between the matrix and reinforcement particles with the effect of increasing pressure. The quality of structural integrity of the material system and concomitant tribological and other performances of MMCs can vary during different process- and material-induced variations due to multiple phase structures of MMCs [2]. In addition, while the maximum contact zone temperatures measure from 68–71 °C for MMCs produced at 480 and 640 MPa, this range decreases to 60–62 °C in composite materials produced at 820 MPa, as seen in Figure 9c. The realization of the temperatures reached in the contact zone shows that the porous structures in the composite material and the strong interfacial bonding quality between the chips significantly affect the heat conduction ability [62].

The S/N ratio approach is performed to evaluate the temperature differences in the contact zone similar to specific wear rates. However, the opposite tendency is observed for the temperature changes. The first levels of temperature and pressure and unreinforced ones are the most suitable for decreasing temperature in the contact zone. As mentioned in the description of the experimental results, the measured temperature is elevated with increasing reinforcement content due to significantly harder GGG-40 reinforcement than the CuSn10 matrix. Considering the weight loss results in our prior work [28], it is an expected condition that the temperature differences show such a trend since it is more difficult to remove the material from the harder region. As the increasing temperature and pressure provide better structural integrity, it is difficult to erode the metallic chips from the surface due to increasing resistance to the plastic deformation mechanism, causing an increase in temperature in the contact area. However, these influences are quite low compared to the reinforcement ratio effect.

**Figure 10:**
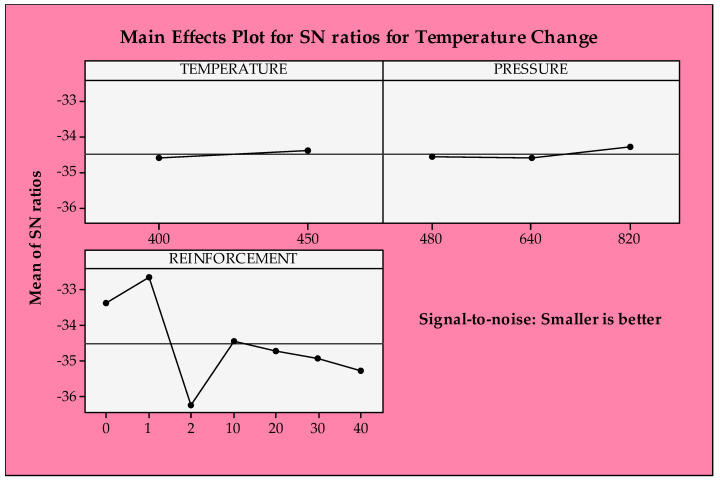
S/N ratios of temperature change.

Tribological characteristics such as surface roughness, weight loss, coefficient of friction, specific wear rate, and temperature differences in the contact zone are crucial for end-product quality and power consumption [69]. However, the effect of these properties on the tribological performance is a highly complex matter. In this context, controlling the part quality can be achieved by assessing and optimizing the tribological properties and other material aspects, such as physical, chemical, and mechanical properties. The mechanics behind the tribological performance are very dynamic, complicated, and confused topics due to various process-induced variations [70]. Hence, it is arduous to evaluate it using experimental and theoretical approaches. However, the source(s), detection, and elimination of these variables need extra attention to monitor microstructural evolution and to determine final product quality. For this reason, a diverse community of a wide range within academia and industry often opts for a “trial and error” way to achieve the desired end-product characteristics. In this regard, to obtain the best specific wear rates and temperature differences, this work was designed and performed both experimentally and statistically. According to Figure 10, optimum parameters for the minimum temperature change are lower temperature, higher pressure, and a pure CuSn10 material system.

### 3.4. ANOVA Evaluation for Temperature Change and Specific Wear Rate

ANOVA provides a critical evaluation approach about the experimental results in engineering applications due to its reliable and broad examples for many areas. Table 4 outlines the ANOVA analysis for specific wear rate and temperature change. Before evaluating the results, it is important to mention here that the total percentage contributions of the two results are 97.8 and 96.9, respectively. This shows that the selected inputs or production parameters are sufficient to determine the important sources of the outputs. In addition, when looking at the statistical values, reinforcement is the most influential parameter on both specific wear rate and temperature changes according to contribution rates. Except for this, temperature and pressure seem ineffective with low percent contributions. Moreover, F values confirm these results, indicating the superiority of reinforcement (248.5 and 166.14). According to P values, reinforcement is significant on both outputs (0.000 < 0.05), and according to temperature changes, temperature (0.01 < 0.05) and pressure (0.002 < 0.05) also have importance. Therefore, the temperature and pressure parameters in the production process of composite materials should be considered for controllable temperature changes.

## 4. Conclusions

In this study, GGG-40-reinforced CuSn10 metal matrix composites were successfully produced by the hot pressing method. Cu-based samples were produced with five different GGG-40 contents (0 wt%, 10 wt%, 20 wt%, 30 wt%, and 40 wt%), three production pressures (820, 640, and 480 MPa), and two temperatures (400 and 450 °C). Two non-waste samples (i.e., pure CuSn10 and pure GGG-40) were also utilized for comparison purposes. Additionally, the tribological performance of produced MMCs was experimentally and statistically assessed, consisting of a parametric optimization point of view utilizing the Taguchi and ANOVA methods. In this regard, specific wear rate and temperature differences in the contact zone, which directly or indirectly affect component life, were examined as outputs while production parameters, namely temperature, pressure, and reinforcement content, were handled as inputs. The experimental outcomes of this study are summarized as follows:

1. The SEM observations show that wear behavior changes depending on the reinforcement content in the MMC system. In the beginning, abrasive wear behavior is observed with decreasing reinforcement content; however, in the following process, the adhesive wear behavior occurs with elevated temperature.
2. Considering both S/N ratio and ANOVA statistical analysis outcomes, it is seen that reinforcement is the most dominant production parameter on specific wear rate and temperature changes in the contact zone with 97.6 and 94.8 total percentage contributions, respectively. Plus, F values verify this situation (248.50 and 166.14, respectively).
3. After the wear test, the maximum temperature is measured as 82 °C in a pure GGG-40 sample. However, the minimum temperature (52 °C) is observed in a pure CuSn10 specimen due to the higher thermal conductivity of the matrix phase. Naturally, an increment in reinforcement content elevates the temperature of the contact zone due to the increased friction coefficient induced by harder GGG-40 chips.
4. A better structural integrity is achieved by increasing production temperature (from 400 to 450 °C) and production pressure (from 480 to 820 MPa). This achieved better structural integrity and increased the resistance to plastic deformation and made it difficult to erode the material from the surface during wear, causing an increase in temperature. On the temperature changes, it should be noted that temperature and pressure seem to be important, along with reinforcement, according to F values.
5. According to the S/N ratio analyses for the specific wear rates, the first temperature level, 400 °C, and the middle range of pressure, 640 MPa, are the best conditions. Considering the influence of reinforcement content, it is observed that pure GGG-40 samples demonstrate the lowest specific wear rate compared to the other samples because the initial iron chips are harder than bronze chips. In addition, a gradually increased reinforcement ratio from 10 to 40 wt% improves wear rate characteristics. After this sample, the 60C40G composite produced with 450 °C and 480 MPa shows the lowest specific wear rate.
6. As seen from the graphical, statistical, and optimization charts, production parameters exhibit unique importance, and have a determinative effect on the tribological properties. Hence, the selection and application of the proper production parameters are crucial points that should be considered, as they directly or indirectly influence the final product’s performance.
7. The proposed method in this study offers an efficient way to manufacture Cu-based MMCs recycled from waste metallic chips.

## Figures and Tables

**Table 1:** Chemical composition of matrix and reinforcement materials at wt% [28,53].

Materials	C	Si	Mn	S	Mg	P	Cu	Sn	Zn	Pb
**CuSn10**	-	-	-	-	-	-	89.2	9.3	0.41	0.01
**GGG-40**	3.4	2.5	0.13	0.01	0.046	0.08	-	-	-	-

**Table 2:** Composite material production parameters.

Specimen Code	Temperature (°C)	Pressure (MPa)	Mixture Weight Ratio (wt%)
**60C40G**	400, 450	480, 640, 820	%60 CuSn10-%40 GGG-40
**70C30G**	400, 450	480, 640, 820	%70 CuSn10-%30 GGG-40
**80C20G**	400, 450	480, 640, 820	%80 CuSn10-%20 GGG-40
**90C10G**	400, 450	480, 640, 820	%90 CuSn10-%10 GGG-40
**100C**	400, 450	480, 640, 820	%100 CuSn10-%0 GGG-40

**Table 3:** Experimental results.

Experiment Number	Specimen Code	Temperature T (°C)	Pressure P (MPa)	Reinforcement Ratio R (%wt.)	Temperature Change T (°C)	Specific Wear Rate SWR
1	60C40G	400	480	40	60.06	6.33
2	70C30G	400	480	30	57.90	7.97
3	80C20G	400	480	20	56.41	8.51
4	90C10G	400	480	10	54.67	10.62
5	100C	400	480	0	48.96	16.81
6	60C40G	400	640	40	58.61	7.32
7	70C30G	400	640	30	57.03	7.51
8	80C20G	400	640	20	56.22	8.29
9	90C10G	400	640	10	54.29	10.05
10	100C	400	640	0	45.96	15.18
11	60C40G	400	820	40	56.22	6.53
12	70C30G	400	820	30	54.93	8.18
13	80C20G	400	820	20	53.54	8.84
14	90C10G	400	820	10	52.45	11.19
15	100C	400	820	0	47.35	14.67
16	60C40G	450	480	40	58.83	5.69
17	70C30G	450	480	30	56.22	7.05
18	80C20G	450	480	20	53.96	8.89
19	90C10G	450	480	10	50.64	11.59
20	100C	450	480	0	44.96	16.37
21	60C40G	450	640	40	60.16	5.97
22	70C30G	450	640	30	58.38	7.53
23	80C20G	450	640	20	54.90	9.01
24	90C10G	450	640	10	52.90	10.86
25	100C	450	640	0	46	15.02
26	60C40G	450	820	40	53.74	7.76
27	70C30G	450	820	30	50.87	9.60
28	80C20G	450	820	20	51.54	9.88
29	90C10G	450	820	10	51.29	10.81
30	100C	450	820	0	46.09	13.87
31	Pure CuSn10	450	820	* 1	42.83	14.29
32	Pure GGG-40	450	820	* 2	64.77	4.01

* 1 shows the pure bronze, * 2 shows the pure cast iron.

**Table 4:** Analysis of variance for S/N ratios of experimental results.

Source	Degree of Freedom	Sum of Squares	Mean Square	*F*-Value	*p*-Value	Percent Contribution (%)
*Specific Wear Rate*						
Temperature	1	0.057	0.057	0.15	0.706	0.0001
Pressure	2	1.171	0.5856	1.49	0.241	0.2
Reinforcement	6	585.969	97.6615	248.50	0.000	97.6
Residual error	32	12.576	0.3930	-	-	2.2
Total	41	599.773	-	-	-	100
*Temperature Change*						
Temperature	1	0.3886	0.38862	7.53	0.010	0.7
Pressure	2	0.7629	0.37143	7.39	0.002	1.4
Reinforcement	6	51.4356	8.57260	166.14	0.000	94.8
Residual error	32	1.6512	0.05160	-	-	3.1
Total	41	54.2382	-	-	-	100

## Data Availability

The data underlying this article will be shared on reasonable request from the corresponding author.
